# Frequency of *Leishmania* spp. infection among HIV-infected patients living in an urban area in Brazil: a cross-sectional study

**DOI:** 10.1186/s12879-020-05622-2

**Published:** 2020-11-25

**Authors:** M. A. Cunha, B. J. Celeste, N. Kesper, M. Fugimori, M. M. Lago, A. S. Ibanes, L. M. Ouki, E. A. Simões Neto, F. F. Fonseca, M. A. L. Silva, W. L. Barbosa Júnior, J. A. L. Lindoso

**Affiliations:** 1 Department of Infectious Diseases, Federal University of Rio Grande do Norte, Rua Cônego Monte, 110, Quintas, Natal/RN, Rio Grande do Norte 59037-170 Brazil; 2 Department of Infectious and Parasitic Diseases, University of São Paulo Medical School, Sao Paulo, Sao Paulo Brazil; 3 Laboratory of Seroepidemiology and Immunobiology, Institute of Tropical Medicine of São Paulo, Sao Paulo, Sao Paulo Brazil; 4 Laboratory of Protozoology, Institute of Tropical Medicine of São Paulo, Sao Paulo, Sao Paulo Brazil; 5 Outpatient sector, Emilio Ribas Institute for Infectious Diseases, Sao Paulo, Sao Paulo Brazil; 6 Department of Medicine Course, Federal University of Maranhão, Pinheiro, Maranhão Brazil; 7 Department of Diseases of Chronic Conditions and Sexually Transmitted Infections, Ministry of Health of Brazil, Brasília, Federal District Brazil; 8 Parasitology, Aggeu Magalhães Research Center, Recife, Pernambuco Brazil

**Keywords:** Leishmaniasis, Acquired immunodeficiency syndrome, Diagnosis, Prevalence

## Abstract

**Background:**

There is little information about the frequency of *Leishmania* infection in asymptomatic people living with HIV (PLWH) and about the performance of laboratory diagnostic methods in coinfected patients in Latin America. The main objective of this study is to evaluate the frequency of *Leishmania* spp. infection in HIV-infected patients living in an urban area in Brazil.

**Methods:**

To detect *Leishmania* infection, diagnostic tests were performed to detect anti-*Leishmania* antibodies (ELISA using *Leptomonas seymouri* antigens; ELISA using rK39 antigens; ELISA using rK28 antigens; indirect fluorescent-antibody test (IFAT); direct agglutination test (DAT)) and *Leishmania* DNA (polymerase chain reaction (PCR) with the target genes kDNA and ITS-1).

**Results:**

The frequency of at least one positive test was 15%. For ELISA using *Leptomonas* antigens and IFAT, there was an association between CD4+ T lymphocyte counts and test positivity, with a higher positivity of these tests in more immunosuppressed patients (CD4+ T cell count < 200/mm^3^).

**Conclusions:**

According to our data, there was a high prevalence of *Leishmania* spp. infections in this population living with HIV. Although there is the possibility of cross-reaction, some tests that are considered highly specific for the diagnosis of *Leishmania* infection were positive. There was also an association between the positivity of some tests studied and lower values of CD4+ T lymphocytes.

## Background

Leishmaniasis is one of the most common neglected tropical diseases, and approximately 350 million people live in areas where there is a risk of infection [1]. In the Americas, 96% of visceral leishmaniasis (VL) cases occur in Brazil [2]. Considered a rural disease until 1980, VL has spread in recent decades to large cities across the country. There was a decrease in the proportion of cases reported by the northeastern states and an increase in autochthonous transmission in larger cities (populations of > 100,000), including some cities in the state of Sao Paulo, [3] where this study is conducted.

In PLWH, leishmaniasis may be an opportunistic infection, and coinfection has become a problem in several parts of the world, especially in East Africa, Brazil and India. Although atypical cases and more severe cutaneous and mucocutaneous leishmaniasis have been reported in PLWH, visceral leishmaniasis is responsible for the major burden of morbidity and mortality in the context of HIV infection [4]. The spread of HIV and VL has produced an overlap between transmission areas of both diseases; therefore, an increase in the number of cases of coinfection has been observed. Both HIV infection and VL produce immunological disturbances that can enhance their effects if they occur concomitantly, contributing to the unfavourable outcome of both diseases [5, 6]. HIV infection dramatically increases the risk of progression from asymptomatic infection towards VL, and in the presence of coinfection, VL tends to be more severe and manifest atypically. *Leishmania* infection also drives HIV replication, inducing the proliferation and differentiation of HIV-infected human monocytes and inhibiting apoptosis of infected cells [5, 7–9].

In Brazil, a progressive increase in HIV/*Leishmania* coinfection has been reported since the beginning of the 1990s [10]. There is a projected continuous rise, mainly because of the overlapping geographical areas of both diseases with urbanization of visceral leishmaniasis and the propagation of HIV transmission to regions with lower urbanization rates and to small- and medium-sized areas [10].

These data are related to symptomatic visceral leishmaniasis, and there are few studies that include asymptomatic patients when evaluating the prevalence of *Leishmania*/HIV coinfection in Brazil, with values of coinfection ranging from 16 to 20.2% in some endemic areas [11, 12]. Increased lethality and therapeutic failure are frequent in coinfected patients, and most HIV-associated visceral leishmaniasis represents reactivation of a prior subclinical infection [13, 14]. Therefore, it is important to evaluate the magnitude of this problem in other areas where asymptomatic immunosuppressed individuals can progress to symptomatic disease more frequently, with poor therapeutic outcomes and a high rate of relapse [13, 15–17]. Another important point is that PLWH could be asymptomatic carriers of *Leishmania* and only develop symptoms in the presence of severe immunosuppression, maintaining the transmission cycle of transmission in areas without other mammalian reservoirs [18, 19].

This study aimed to estimate the frequency of *Leishmania* infection among PLWH from a major national reference centre for HIV in an urban area based on the detection of anti-*Leishmania* antibodies and *Leishmania* DNA.

## Methods

### Participants

The study was conducted at the Institute of Infectious Diseases Emilio Ribas, Sao Paulo, Brazil, from April 2015 to March 2016. This institute is a resource for the treatment of HIV infection and infectious tropical diseases in Brazil and assists 8500 PLWH yearly. The included patients were older than 18 years and had a definitive diagnosis of HIV infection according to criteria established by the Ministry of Health of Brazil [20]. Severe immunodeficiency was recorded if the patient had an AIDS-defining illness or CD4+ T lymphocyte count < 200 cells/mm^3^ at the time of inclusion.

Exposure to an endemic area of VL was recorded if the patient had been born in or lived for more than 1 year in a municipality with autochthonous transmission of VL, as reported by the National Surveillance System of the Ministry of Health [21]. Personal, epidemiological, clinical and laboratory data were obtained by the analysis of clinical records. Written, free-and-informed consent to participate in the study was obtained from all individuals.

### Laboratory tests performed

Sample blood was obtained from peripheral veins and used to detect *Leishmania* DNA (kDNA and ITS-1) and anti-*Leishmani*a antibodies by ELISA using different antigens (recombinant K39, recombinant K28, *Leptomonas seymouri* antigens), indirect fluorescent antibody test (IFAT) and direct agglutination test (DAT).

#### Serological methods

##### *Leptomonas* ELISA

*Leptomonas seymouri* antigenic extract was prepared as previously described [22, 23], with some modifications. Microtiter plates (Corning Incorporated, NY, USA) were coated with 50 μL of parasite antigens (4 μg/mL) diluted in carbonate-bicarbonate buffer (0.05 M, pH 9.6) overnight at 4 °C. The plates were blocked with 0.01 M phosphate buffered saline (pH 7.2) with 0.05% Tween-20 and 5% fat-free milk for 30 min at room temperature. The plates were incubated with 50 μl of diluted human antibody (1:200) for 1 h at 37 °C. The plates were washed five times and incubated with peroxidase-conjugated goat anti-human IgG diluted in PBS (1:2.000) for 1 h at room temperature. The plates were washed, hydrogen peroxide and O-phenylenediamine dihydrochloride (OPD-tablets, Sigma Co) were added to each well and incubated for 30 min at 37 °C in the dark, and the reaction was stopped by adding 25 μL of 4 N HCl. The absorbance at 492 nm was measured using a spectrophotometer (Titertek Multisplan Plus, Helsinki, Finland). The cut-off was determined by the value that demonstrated better sensitivity and specificity in the receiver operating characteristic (ROC) curve including the positive and negative controls. All experiments were independently repeated at least twice.

##### rK39 ELISA and rK28 ELISA

rK39 and rK28 were provided by the Infectious Disease Research Institute, Seattle, WA, and the ELISA was carried out as previously described. Briefly, flat-bottom 96-well microtiter plates were coated with 50 ng/well (100 μl) of rK28 and rK39 antigen in coating buffer and incubated overnight at 4 °C. Plates were blocked with blocking solution (5% fat-free milk (Molico, Nestlé) in 0.05 M phosphate buffer (PBS) containing 0.1% Tween-20) for 2 h at 37 °C. Plates were loaded with serum samples diluted in buffer solution (1:100) and incubated for 30 min at 37 °C. The plates were washed and incubated with peroxidase-conjugated goat anti-human IgG (1:30.000 dilution in blocking solution) at 37 °C for 30. The plates were incubated with TMB substrate (Sigma, Co) for 7 min in the dark, and the reaction was stopped with 25 μL of 2 N H2SO4. Optical density was measured at 450 nm. The reactivity index (RI) was calculated for each sample by dividing the sample absorbance value by the cut-off. Samples were considered positive if the RI value was ≥1.

##### IFAT

The test was performed according to the protocol described by Guimaraes and others, 1974 [24]. Briefly, slides were coated with *L. major-*like promastigote antigens (MHOM/BR/71/49). Human sera were added and incubated for 30 min at 37 °C. Fluorescence was evaluated by fluorescence microscopy.

##### DAT

A kit produced at the Prince Leopold Institute of Tropical Medicine (Amsterdam, Netherlands) was used according to the manufacturer’s instructions for serum samples, according to Harith and collaborators [25]. The cut-off value was set at > 1:6.400.

### Molecular methods

#### DNA extraction

Extraction of DNA from blood was performed using the QIAamp Mini Kit (QIAGEN, Chatsworth, CA, USA) according to the manufacturer’s instructions.

#### kDNA PCR

The kDNA region (720 bp) was amplified as described elsewhere by Aransay et al., 2000. Briefly, extracted DNA was amplified using the primers LINR4 and LIN19. After preheating at 95 °C for 1 min, the samples were subjected to 30 amplification cycles in the following sequence: each cycle consisted of 30 s at 95 °C, 30 s at 55 °C, and 1 min at 70 °C, and an extra elongation step (5 min at 72 °C) was performed [26]. Amplification was determined using electrophoresis on a 1% agarose gel, and the amplified sequences were visualized by ethidium bromide staining.

#### ITS-1 PCR

The ITS-1 region was amplified using primers LITSR and L5.8S described elsewhere [27]. PCR was performed according to the manufacturer’s instructions using GoTaq® Hot Start Green Master Mix Protocol (Promega Corporation, Madison, USA) for 30 cycles as follows: 1 cycle of 3 min at 95 °C; 35 cycles of 40 s at 95 °C, 45 s at 53 °C, 1 min at 72 °C and 6 min for final extension at 72 °C. DNA amplification was determined by electrophoresis on a 2% agarose gel and visualized by ethidium bromide staining.

### Statistical analysis

A database was created in Microsoft Excel® 2013. The analysis and interpretation of the results were performed using Microsoft Excel® 2013 and Graph Prism® 5.

The groups “any test positive” versus “all tests negative” were compared according to the following characteristics: age, sex, presence of epidemiology for VL, time since diagnosis of HIV infection, CD4 + T lymphocyte count, regular use of antiretroviral therapy (ART), history of previous opportunistic infection (including VL), presence of comorbidities, presence of characteristic symptoms of VL, presence of clinical complications of treatment at the time of inclusion, and presence of laboratory alterations (anaemia, leukopenia or thrombocytopenia). Participants were actively questioned about the diagnosis of Chagas disease because of the possibility of cross reactivity in some methods. Additionally, the same comparison was performed for each test separately.

The “p” value was calculated using different statistical tests, depending on the analysed variable: Chi-square test and Fisher’s exact test for categorical variables; Mann-Whitney test for continuous variables with non-parametric distribution; and t test for continuous variables with parametric distribution. For continuous variables with a normal distribution, the mean values were used for comparison purposes; when the variables had a nonparametric distribution, the median values were used. Data with *p* values < 0.05 were considered statistically significant. The agreement between the tests was calculated using the kappa index, with the following interpretations: values < 0 - none; values between 0.00 and 0.19 - poor; values between 0.20 and 0.39 - reasonable; values between 0.40 and 0.59 - moderate; values between 0.60 and 0.79 - excellent; and a value of 1.0 - perfect.

## Results

From April 2015 to March 2016, 245 participants were included in this study; however, five of them were later excluded due to problems related to storage of samples. Therefore, 240 individuals were included in the analysis. Participants’ ages ranged from 24 to 80 years, with a mean age of 45.5 years, and men comprised the majority of the samples (172, 71.6%). The mean time between HIV-infection diagnosis and study inclusion was 13.3 years. The viral load ranged from nondetectable to 3.206.948 copies/mL, and the mean CD4+ T lymphocytes were 564.4 cells/mm^3^ (ranging from 2 to 1.724 cells/mm^3^). Regarding the use of HIV therapy, 87.9% (211/240) of participants were on regular ART. Ninety-two (38.3%) individuals had a history of exposure to endemic areas of VL. The baseline characteristics of the sample population are shown in Table 1.

**Table 1:** Distribution of the 240 participants according to by age, sex, clinical, epidemiological and laboratory parameters

	**Mean (range)**
Age (years)	46 (24–80)
Time since HIV diagnosis (years)	13.3 (1–30)
TCD4+ (cells/mm^3^)	568.4 (2–1724)
	**Frequency – n (%)**
Undetectable viral load	195 (81.3)
Male (%)	172 (71.6)
On regular ART (%)	211 (87.9)
Previous exposure to endemic area for VL	92 (38.3)

No individual had visceral or cutaneous leishmaniasis previously, and one patient had Chagas disease (undetermined form). Twenty-eight participants were in treatment for at least one disease, and 17 of them had at least one sign/symptom (fever and/or splenomegaly and/or weight loss) considered compatible with visceral leishmaniasis diagnosis, according to WHO [1]. In addition to these patients, one patient had splenomegaly, totalling 18 individuals with at least one sign/symptom considered compatible with VL. At the discretion of the attending medical staff, two of the patients were submitted to bone marrow aspiration to search for *Leishmania,* and in both cases, the result was negative. Therefore, no patient had clinically confirmed leishmaniasis.

The diseases being treated at the moment of inclusion and the number of times that each disease was present were as follows: tuberculosis (9), cytomegalovirus disease (5), cerebral toxoplasmosis (3), lymphoma (3), Kaposi sarcoma (2), cryptococcal meningitis (2), neurosyphilis (2), muscular abscess (1), CNS demyelinating disease (1), pulmonary aspergillosis (1), hepatic cirrhosis (1) and pneumocystosis (1). It is noteworthy that four participants had more than one disease concomitantly.

Table 2 shows the positivity of each diagnostic test. Among the 240 included participants, nine were positive by *Leptomonas* ELISA (3.75%), three by rK39 ELISA (1.25%), six by rK28 ELISA (2.5%), 11 by IFAT (4.5%), four by kDNA PCR (1.66%) and 10 by ITS-1 PCR (4.16%). No sample was positive by DAT. Considering those participants who had at least one positive test as infected, the estimated prevalence of asymptomatic infection was 15% (36 participants).

**Table 2:** Number and percentage of positive tests for detection of anti-Leishmania antibodies and detection of *Leishmania ssp* DNA from 240 participants

	Total n (%)	95%CI
**ELISA** ***Leptomonas***	9 (3.8)	2.0–7.0
**ELISA rK39**	3 (1.3)	0.4–3.6
**ELISA rK28**	6 (2.5)	1.2–5.3
**IFAT**	11 (4.6)	2.6–8.0
**kDNA PCR**	4 (1.7)	0.6–4.2
**ITS-1 PCR**	10 (4.2)	2.3–7.5
**At least one positive test**	36 (15)	10.7–20.2

*CI* confidence interval

Comparing all tests, concordance between tests was poor (kappa test < 0.20). No patient had two positive molecular methods, but one ITS-1 PCR-positive patient had positive serological methods, positive by *Leptomonas* ELISA and IFAT.

Patients with infection by *Leishmania* spp., defined as positivity by any test, showed similar distribution by age, sex, presence of signs/symptoms, exposure to endemic areas to VL, previous opportunistic diseases, regular ART use, and comorbidities, with no statistically significant association between the positivity of the tests and these factors. For CD4+ T cell counts, there was a significant association between at least one test and CD4+ T cell count less than 200 cells/mm^3^ (Table 3). The same variables were evaluated considering each test separately, also with no associations (data not shown) except for CD4+ T cell count: there was a significant association between *Leptomonas* ELISA and IFAT and the presence of CD4+ T cell count less than 200 cells/mm^3^ (Table 4). For the other methods, this association was not demonstrated (data not shown).

**Table 3:** Distribution of tests used to detect infection by *Leishmania ssp*. in 240 participants. The positive and negative results (“any test positive” versus “all tests negative”) were evaluated according to age, sex, exposure to endemic areas of VL, time of HIV diagnosis, ART use, previous opportunistic infection, comorbidities and laboratorial data

	Any test positive	All tests negative	p	OR	CI (95%)
**Age, mean**	50,9	49,7	0,37*		
**Sex, n**					
Male	27	146	0,67**	1,19	0,53 - 2,67
Female	9	58			
**Exposure to endemic areas of VL**					
Yes	15	77	0,65**	1,18	0,47 - 1,60
No	21	127			
**Time of HIV diagnosis, median**	16,5	14,5	0,27***		
**T CD4+ (cells/ml), median**	561,0	547,5	0,51***		
**T CD4+ (cells/ml)**					
< 200	8	19	0,03**	2,78	1,11 - 6,96
≥ 200	28	185			
**Regular ART use**					
Yes	29	182	0,14**	0,5	0,84 - 3,64
No	7	22			
**Previous opportunistic infection, n**					
Yes	6	47	0,40**	0,67	0,26 - 1,70
No	30	157			
**Comorbidities, n**					
Yes	14	95	0,40**	0,73	0,35 - 1,50
No	22	109			
**Presence of signal/symptoms compatible with visceral leishmaniasis**					
Yes	3	15	0,74****	1,15	0,31 - 4,18
No	33	189			
**Anemia and/or leukopenia and/or thrombocytopenia, n**					
Yes	7	51	0,47**	0,72	0,30 - 1,75
No	29	153			

*Student’s T test; **Qui-squared test; *** Mann–Whitney U test; ****Fisher’s exact test

**Table 4:** ELISA Leptomonas and IFAT positivity related to TCD4 + lymphocyte count, categorized by values greater than or equal to and less than or equal to 200 cells / mm^3^

	Positive n (%)	Negative n (%)	*p* - value
**ELISA** ***Leptomonas***			
TCD4 + < 200	4 (14.8)	23 (85.2)	0.011*
TCD4 + ≥200	5 (2.3)	208 (97.7)	
**IFAT**			
TCD4 + < 200	5 (18.5)	22 (81.5)	0.004 *
TCD4 + ≥200	6 (2.8)	207 (97.2)	

* Fisher exact test applied

## Discussion

This study showed a frequency of 15% of *Leishmania* spp. infection in PLWH, with weak concordance of the tests utilized. There are few studies evaluating the prevalence of *Leishmania* asymptomatic infection among PLWH, and there are no studies analysing this frequency in an urban area with no reports of autochthonous transmission of VL in Brazil. Taking into account the overall prevalence (at least one positive test) observed in this study, the result was less than that reported by Orsini et al., 20.2% [12]. In another study performed in Brazil, Carranza-Tamayo et al. showed similar data, with an overall prevalence of 16.0% [11]. Both studies were conducted in another area of the country (Federal District) and did not include data from Sao Paulo. There are no studies including patients from the Southeast Region.

Considering studies in other parts of the world, the frequency of coinfection is lower than that found by Garrote et al., 64.0%, in Spain [28]. These discrepancies are probably explained by the use of different diagnostic tests, including different antigens and molecular targets. Recently, in a systematic review of asymptomatic visceral leishmaniasis in the Indian subcontinent, Hirve et al. [29], including 31 articles with different tests (rK39 immunochromatographic test, rK39 ELISA, DAT, PCR or Leishmanin skin test), found a prevalence from 0.25 to 36.9%, depending on the method used. Recently, Echchakery et al. [18], in Morocco, found a prevalence of 5% in PLWH by indirect immunofluorescence. Clearly, there is no consensus about the best diagnostic tool to estimate the frequency of asymptomatic *Leishmania* infection in the population, and in the context of HIV infection, even in fully developed symptomatic disease, there are few data from Latin America regarding the performance of diagnostic methods [30].

The data also demonstrated poor agreement between serological tests. This fact has been shown by other prevalence studies involving PLWH and immunocompetent individuals [11, 12, 31, 32] and can be accounted for by the use of different antigens and molecular targets (surface, soluble or recombinant antigens) that can detect many stages of asymptomatic infection.

ELISA using *Leptomonas seymouri* antigen was performed for the first time in people with HIV infection in this study. It has already been demonstrated that this antigen has good performance for the diagnosis of visceral leishmaniasis; in fact, its performance is comparable to that of the *L. chagasi* antigen. Similar to other crude antigens, there is the possibility of cross-reactivity with *T. cruzi* and other *Leishmania* species [22]. Recently, Kesper et al. [33], while performing ELISA using *Leptomonas seymouri* and *Crithidia fasciculata* antigens, showed 100% reactivity with sera from visceral leishmaniasis (VL) cases and no reactivity with American tegumentary leishmaniasis (ATL). Nevertheless, these individuals did not have HIV infection. In this study, one patient had Chagas disease (undetermined form), and his serum was positive for *Leptomonas* antigen. We strongly suggest that further studies are necessary to determine the sensitivity and specificity for VL diagnosis in individuals with HIV infection using the *Leptomonas* antigen.

IFAT using *L. major*-like antigen is a test recommended by the Ministry of Health of Brazil for the diagnosis of leishmaniasis [34]. In this study, IFAT showed greater positivity compared to *Leptomonas* ELISA*.* In other studies evaluating the prevalence of *Leishmania/*HIV coinfection, ELISA using crude antigens demonstrated greater positivity compared to IFAT [11, 12], and this was attributed to the fact that IFAT detects mainly antibodies against surface antigens, while crude-antigen ELISA detects a wider variety of antibodies directed against soluble antigen components. In our study, this difference was not observed (Table 2) and was probably related to divergence between the specificity of these tests and the immunological characteristics of antigens.

Considering only serological methods, we found that a lower CD4+ T cell count was associated with a positive ELISA for *Leptomonas* or IFAT. These data can be, at first, considered surprising, given that serological methods have worse sensitivity for visceral leishmaniasis diagnosis in PLWH compared to immunocompetent individuals [8, 30, 35]. This concept is applicable to other infectious diseases, especially to the advanced stage of AIDS, in which severe T and B lymphocyte dysfunction triggers decreased production of specific antibodies [5]. It is important to highlight that all this information is related to the performance of diagnosis tests in subjects with symptomatic visceral leishmaniasis, and herein, we discuss asymptomatic individuals. Another important point is the possibility of cross-reaction of these tests. Although there was no association between current disease in treatment and test positivity, it is possible that these individuals with lower values of CD4+ T cell counts had subclinical or latent diseases, which may have interfered with the results. Another question is the possible variability of humoral response in PLWH. Gradoni et al. [36], comparing serological IFAT results of HIV-infected and non-HIV-infected individuals with visceral leishmaniasis, showed worse sensitivity of this method in those infected with HIV and a greater variability of results in this group, with some people with IgG levels far above the non-HIV-infected subjects. These findings may reflect the temporal sequence of acquisition of the two infectious agents (HIV and *Leishmania*). Individuals who acquire *Leishmania* infection before HIV infection should produce high levels of IgG directed against antigens recognized before T cell impairment due to HIV. This response is probably related to increased IgG production because of nonspecific polyclonal B cell activation, which occurs more frequently in people with severe immunosuppression [5, 36]. This abnormal humoral response, more evident in severely immunosuppressed patients, can explain a greater proportion of positive serological tests in the group, considering that all samples are from individuals currently living in a non-endemic area for VL that probably acquired *Leishmania* infection before HIV infection.

rK39 ELISA had the lowest positivity among all serological methods. This antigen has been used both in ELISA and in rapid diagnostic tests [37–39], including in PLWH [40, 41]. As rK39 is characteristically associated with active disease [42, 43], a sample composed predominantly of asymptomatic individuals is expected to have a lower positivity. Furthermore, even considering the diagnosis of symptomatic disease, the sensitivity of ELISA using the rK39 antigen is considerably worse in PLWH [44]. Therefore, although it can be possible to detect this antigen in asymptomatic individuals [38], these characteristics can limit its use in this context. In the general population, the sensitivity and specificity of recombinant K28 antigen (99.6% and 95–100%, respectively) is similar to rK39 ELISA for the diagnosis of VL [45]. rK28 antigen can be used in rapid tests with good performance, showing high specificity (near 100%) and sensitivity (92%) for the diagnosis of visceral leishmaniasis [46]. However, it has not been used in studies including people from Latin America, and there are few studies discriminating its performance in PLWH. Silva et al. [47], using two immunochromatographic tests to detect the antibodies anti-rK39 and anti-rK28, found sensitivities of 67.74 and 61.29%, respectively, in a group with active visceral leishmaniasis and HIV infection, showing poor performance when compared with active visceral leishmaniasis without HIV infection. In our study, in a group of asymptomatic individuals, rK28 ELISA positivity was greater than rK39 ELISA positivity, which can suggest a good perspective of its use in HIV-infected Latin American asymptomatic patients, perhaps as an early marker of *Leishmania* infection.

In our sample, no individual had a positive DAT. This test is one of the most widely used diagnostic tests for VL around the world [48], and in comparison with other serological methods, it has a good performance in PLWH [30]. DAT can be used to diagnose active forms of the disease and can diagnose infections before the clinical presentation, with results declining to negative values 1 year after the cure [49]. There are two important points to highlight about the population included in this study. First, the individuals included were predominantly asymptomatic, and nobody had a confirmed leishmaniasis diagnosis or a previous history of this disease. The performance of this test considering this scenario had never been evaluated, probably because there is no gold standard to compare this test with other tests and to establish sensitivity and positivity. Moreover, although a part of the cohort had lived in endemic areas in the past, all subjects were currently living in a non-endemic area for VL. We believe the fact that we are analysing a sample composed of asymptomatic HIV-infected patients not continuously exposed to *Leishmania* can explain this result, as the positivity of the test depends on the presence of active disease, recent cure or exposure to antigen.

Regarding molecular methods, we used two different targets to perform polymerase chain reaction (PCR). In a systematic review and meta-analysis, de Ruiter et al. [50] found a pooled sensitivity and specificity of PCR in peripheral blood of 93.1 and 95.6%, respectively. There are studies demonstrating promising results in immunosuppressed patients as well [51, 52]. Although kDNA is more commonly used for DNA amplification of *Leishmania* because of the high number of copies per parasite [53], in this study, ITS-1 PCR had greater positivity (4.2%) compared to kDNA PCR (1.7%). Several different genomic targets are used for *Leishmania* spp. detection, and there is no consensus about the best one, especially due to the discrepancies of objectives and methods of each study [53]. The fact that no patient was positive by PCR and the discrepancy between the two tests reinforces this limitation and the necessity of further studies about this theme, including symptomatic and asymptomatic subjects. Molecular methods have been developed with the purpose of complementing and creating alternatives for the diagnosis of leishmaniasis, as well as the possibility of follow-up and laboratory confirmation of cure [53, 54]. It is possible that more immunosuppressed individuals present a greater periodicity of parasitic circulation in their body. The higher positivity of this method in this type of patients may indicate that this target is more effective in identifying intermittent parasitaemia in this group of patients and that PCR can be used in the future to screen individuals at risk of developing manifest leishmaniasis. This information would be of extreme importance for these individuals with advanced immunosuppression, who are known to present with more severe forms of the disease, more complications and higher mortality [55, 56].

The presence of signs/symptoms did not demonstrate an association with test positivity. First, information about cities with autochthonous transmission was obtained through records of the Ministry of Health of Brazil, and a considerable portion of these cities were considered to have autochthonous transmission due to the presence of just one or a few cases of confirmed VL. Therefore, many participants may not have been exposed to VL, despite living in transmission areas. In the same way, it was not possible to guarantee that, at the moment the individual lived in an endemic area, autochthonous VL was present. Most cases of HIV infection in Brazil are located in the South and Southeast Region, where Sao Paulo state is located. Regarding VL, between 1999 and 2013, 2.328 autochthonous cases of visceral leishmaniasis were confirmed in Sao Paulo state, corresponding to 80 cities with VL transmission. Of these, 202 patients died, corresponding to a lethality of 8.7%. Of note, Sao Paulo city has had no report of autochthonous VL transmission to date [57].

Injecting drug use is the major cause of HIV infections in *Leishmania/*HIV coinfection in many parts of the world [1]. In Brazil, this pathway of HIV transmission is not as common, and sexual transmission corresponds to more than 95% of HIV infection acquisition [58]. Thus, although we do not have data about the percentage of injecting drug users in the sample, we consider the possibility of *Leishmania* acquisition through this route to be very low. Concerning the presence of symptoms, 17 of 28 participants presenting with a symptom defined as suggestive of VL had other diseases, including tuberculosis as the most common. Therefore, some of these symptoms were probably more attributable to these diseases than to the diagnosis of VL or to other diseases not yet diagnosed. Therefore, there was no association with this variable and positivity of diagnostic tests.

## Conclusions

We showed a prevalence of 15% of *Leishmania* spp. infection in PLWH. These data should be taken with caution. It is important to highlight that serology at a given time may reflect contact with parasites followed by sterile cure. Considering only molecular methods, the prevalence falls to 5.8%. Nevertheless, the natural history of *Leishmania* infection in humans is dynamic, with parasites present in peripheral blood at a given time and with immune responses acting to inhibit parasite production at another time, a fact that can be more evident in the context of HIV infection. Thus, positivity of indirect tests may reflect an asymptomatic infection, especially considering the possibility of reactivation in HIV immunosuppressed patients. The weak overlap observed here and in other studies indicates that the use of more than one diagnostic method may increase the proportion of cases detected. Taking into account the positivity of these tests, including molecular methods and very specific serological tests in individuals with HIV infection living in a city with no autochthonous cases of VL, but in a country with high transmission of VL, it is important to consider leishmaniasis infection as a cause of fever and other symptoms, especially in the context of advanced immunosuppression.

## Data Availability

The datasets used and/or analyzed during the current study are available from the corresponding author on reasonable request.

## Abbreviations

ATL: American tegumentary leishmaniasis
DAT: Direct agglutination test
ELISA: Enzyme-linked immunosorbent assay
IFAT: Indirect fluorescent-antibody test
ITS-1: Internal transcribed spacer 1
kDNA: Kinetoplastid DNA
PCR: Polymerase chain reaction
RI: Reactivity index
VL: Visceral leishmaniasis

## References

[1] WHO: World Health Organization (2010). Expert Committee on the Control of the Leishmaniases & World Health Organization. Control of the leishmaniases: report of a meeting of the WHO Expert Commitee on the Control of Leishmaniases.

[2] Alvar J, Vélez ID, Bern C, Herrero M, Desjeux P, Cano J, Jannin J, Boer MD, the WHOLCT (2012). Leishmaniasis worldwide and global estimates of its incidence. PLoS One.

[3] Werneck GL (2014). Visceral leishmaniasis in Brazil: rationale and concerns related to reservoir control. J Rev Saude Publica.

[4] Jarvis JN, Lockwood DN (2013). Clinical aspects of visceral leishmaniasis in HIV infection. Curr Opin Infect Dis.

[5] Alvar J, Canavate C, Gutierrez-Solar B, Jimenez M, Laguna F, Lopez-Velez R, Molina R, Moreno J (1997). Leishmania and human immunodeficiency virus coinfection: the first 10 years. Clin Microbiol Rev.

[6] Lindoso JA, Cota GF, da Cruz AM, Goto H, Maia-Elkhoury AN, Romero GA, de Sousa-Gomes ML, Santos-Oliveira JR, Rabello A (2014). Visceral leishmaniasis and HIV coinfection in Latin America. PLoS Negl Trop Dis.

[7] Mock DJ, Hollenbaugh JA, Daddacha W, Overstreet MG, Lazarski CA, Fowell DJ, Kim B (2012). Leishmania induces survival, proliferation and elevated cellular dNTP levels in human monocytes promoting acceleration of HIV co-infection. PLoS Pathog.

[8] van Griensven J, Diro E (2012). Visceral leishmaniasis. Infect Dis Clin N Am.

[9] van Griensven J, Zijlstra EE, Hailu A (2014). Visceral Leishmaniasis and HIV Coinfection: time for concerted action. PLoS Negl Trop Dis.

[10] Brazil: Ministry of Health (2011). Department of Health Surveillance. Leishmaniosis history and epidemiological trends and current situation. Recommendations for diagnosis, treatment and follow-up of patients with HIV-Leismania co-infection [in Portuguese].

[11] Carranza-Tamayo CO, de Assis TS, Neri AT, Cupolillo E, Rabello A, Romero GA (2009). Prevalence of Leishmania infection in adult HIV/AIDS patients treated in a tertiary-level care center in Brasilia, Federal District, Brazil. Trans R Soc Trop Med Hyg.

[12] Orsini M, Canela JR, Disch J, Maciel F, Greco D, Toledo A, Rabello A (2012). High frequency of asymptomatic Leishmania spp. infection among HIV-infected patients living in endemic areas for visceral leishmaniasis in Brazil. Trans R Soc Trop Med Hyg.

[13] Morales MA, Cruz I, Rubio JM, Chicharro C, Canavate C, Laguna F, Alvar J (2002). Relapses versus reinfections in patients coinfected with Leishmania infantum and human immunodeficiency virus type 1. J Infect Dis.

[14] Murray HW, Berman JD, Davies CR, Saravia NG (2005). Advances in leishmaniasis. Lancet (London, England).

[15] Cota GF, de Sousa MR, Rabello A (2011). Predictors of visceral leishmaniasis relapse in HIV-infected patients: a systematic review. PLoS Negl Trop Dis.

[16] Coura-Vital W, Araujo VE, Reis IA, Amancio FF, Reis AB, Carneiro M (2014). Prognostic factors and scoring system for death from visceral leishmaniasis: an historical cohort study in Brazil. PLoS Negl Trop Dis.

[17] Pasquau F, Ena J, Sanchez R, Cuadrado JM, Amador C, Flores J, Benito C, Redondo C, Lacruz J, Abril V (2005). Leishmaniasis as an opportunistic infection in HIV-infected patients: determinants of relapse and mortality in a collaborative study of 228 episodes in a Mediterreanean region. Eur J Clin Microbiol Infect Dis.

[18] Echchakery M, Nieto J, Boussaa S, El Fajali N, Ortega S, Souhail K, Aajly H, Chicharro C, Carrillo E, Moreno J (2018). Asymptomatic carriers of Leishmania infantum in patients infected with human immunodeficiency virus (HIV) in Morocco. Parasitol Res.

[19] Ferreira GR, Castelo Branco Ribeiro JC, Meneses Filho A, Dejesus Cardoso Farias Pereira T, Parente DM, Pereira HF, Carlos da Silva J, Zacarias DA, Vieira da Silva L, Medeiros Faustino SK (2018). Human competence to transmit leishmania infantum to lutzomyia longipalpis and the influence of human immunodeficiency virus infection. Am J Trop Med Hyg.

[20] Brazil: Ministry of Health (2018). Technical manual for the diagnosis of HIV infection in adults and children [in Portuguese].

[21] DATASUS: Ministry of Health of Brazil. 2019. Available from: https://datasus.saude.gov.br/. Acessed 01 May 2019.

[22] Ferreira LR, Kesper N, Teixeira MM, Laurenti MD, Barbieri CL, Lindoso JA, Umezawa ES (2014). New insights about cross-reactive epitopes of six trypanosomatid genera revealed that Crithidia and Leptomonas have antigenic similarity to L. (L.) chagasi. Acta Trop.

[23] Umezawa ES, Nascimento MS, Kesper N, Coura JR, Borges-Pereira J, Junqueira AC, Camargo ME (1996). Immunoblot assay using excreted-secreted antigens of Trypanosoma cruzi in serodiagnosis of congenital, acute, and chronic Chagas' disease. J Clin Microbiol.

[24] Guimaraes MC, Giovannini VL, Camargo ME (1974). Antigenic standardization for mucocutaneous leishmaniasis immunofluorescence test. Rev Inst Med Trop Sao Paulo.

[25] el Harith A, Kolk AH, Leeuwenburg J, Muigai R, Huigen E, Jelsma T, Kager PA (1988). Improvement of a direct agglutination test for field studies of visceral leishmaniasis. J Clin Microbiol.

[26] Aransay AM, Scoulica E, Tselentis Y (2000). Detection and identification of Leishmania DNA within naturally infected sand flies by seminested PCR on minicircle kinetoplastic DNA. Appl Environ Microbiol.

[27] Schonian G, Nasereddin A, Dinse N, Schweynoch C, Schallig HD, Presber W, Jaffe CL (2003). PCR diagnosis and characterization of Leishmania in local and imported clinical samples. Diagn Microbiol Infect Dis.

[28] Garrote JI, Gutierrez MP, Izquierdo RL, Duenas MA, Zarzosa P, Canavate C, El Bali M, Almaraz A, Bratos MA, Berbel C (2004). Seroepidemiologic study of Leishmania infantum infection in Castilla-Leon, Spain. Am J Trop Med Hyg.

[29] Hirve S, Boelaert M, Matlashewski G, Mondal D, Arana B, Kroeger A, Olliaro P (2016). Transmission dynamics of visceral leishmaniasis in the Indian subcontinent - a systematic literature review. PLoS Negl Trop Dis.

[30] Cota GF, de Sousa MR, Demarqui FN, Rabello A (2012). The diagnostic accuracy of serologic and molecular methods for detecting visceral leishmaniasis in HIV infected patients: meta-analysis. PLoS Negl Trop Dis.

[31] de Gouvea VL, de Assis TS, Orsini M, da Silva AR, de Souza GF, Caligiorne R, da Silva AC, Peruhype-Magalhaes V, Marciano AP, Martins-Filho OA (2008). Combined diagnostic methods identify a remarkable proportion of asymptomatic Leishmania (Leishmania) chagasi carriers who present modulated cytokine profiles. Trans R Soc Trop Med Hyg.

[32] Moreno EC, Melo MN, Lambertucci JR, Serufo JC, Andrade AS, Antunes CM, Genaro O, Carneiro M (2006). Diagnosing human asymptomatic visceral leishmaniasis in an urban area of the State of Minas Gerais, using serological and molecular biology techniques. Rev Soc Bras Med Trop.

[33] Kesper N, Teixeira MMG, Lindoso JAL, Barbieri CL, Umezawa ES (2017). Leptomonas seymouri and Crithidia fasciculata exoantigens can discriminate human cases of visceral leishmaniasis from American tegumentary leishmaniasis ones. Rev Inst Med Trop Sao Paulo.

[34] Brazil: Ministry of Health (2014). Visceral leishmaniasis surveillance and control manual [in Portuguese].

[35] Elmahallawy EK, Sampedro Martinez A, Rodriguez-Granger J, Hoyos-Mallecot Y, Agil A, Navarro Mari JM, Gutierrez Fernandez J (2014). Diagnosis of leishmaniasis. J Infect Dev Ctries.

[36] Gradoni L, Scalone A, Gramiccia M (1993). HIV-Leishmania co-infections in Italy: serological data as an indication of the sequence of acquisition of the two infections. Trans R Soc Trop Med Hyg.

[37] Diro E, Lynen L, Assefa M, Takele Y, Mengesha B, Adem E, Mohammed R, Kimutai R, Hailu A, Boelaert M (2015). Impact of the use of a rapid diagnostic test for visceral leishmaniasis on clinical practice in Ethiopia: a retrospective study. PLoS Negl Trop Dis.

[38] Nascimento Mdo D, Souza EC, da Silva LM, Leal Pda C, Cantanhede Kde L, Bezerra GF, Viana GM (2005). Prevalence of infection by Leishmania chagasi using ELISA (rK39 and CRUDE) and the Montenegro skin test in an endemic leishmaniasis area of Maranhao, Brazil. Cad Saude Publica.

[39] Peruhype-Magalhaes V, Machado-de-Assis TS, Rabello A (2012). Use of the Kala-Azar Detect(R) and IT-LEISH(R) rapid tests for the diagnosis of visceral leishmaniasis in Brazil. Mem Inst Oswaldo Cruz.

[40] Barbosa Junior WL, Ramos de Araujo PS, Dias de Andrade L, Aguiar Dos Santos AM, Lopes da Silva MA, Dantas-Torres F, Medeiros Z (2015). Rapid tests and the diagnosis of visceral leishmaniasis and human immunodeficiency virus/acquired immunodeficiency syndrome coinfection. Am J Trop Med Hyg.

[41] Cota GF, de Sousa MR, de Freitas Nogueira BM, Gomes LI, Oliveira E, Assis TS, de Mendonca AL, Pinto BF, Saliba JW, Rabello A (2013). Comparison of parasitological, serological, and molecular tests for visceral leishmaniasis in HIV-infected patients: a cross-sectional delayed-type study. Am J Trop Med Hyg.

[42] Badaro R, Benson D, Eulalio MC, Freire M, Cunha S, Netto EM, Pedral-Sampaio D, Madureira C, Burns JM, Houghton RL (1996). rK39: a cloned antigen of Leishmania chagasi that predicts active visceral leishmaniasis. J Infect Dis.

[43] Braz RF, Nascimento ET, Martins DR, Wilson ME, Pearson RD, Reed SG, Jeronimo SM (2002). The sensitivity and specificity of Leishmania chagasi recombinant K39 antigen in the diagnosis of American visceral leishmaniasis and in differentiating active from subclinical infection. Am J Trop Med Hyg.

[44] Moreno EC, Goncalves AV, Chaves AV, Melo MN, Lambertucci JR, Andrade AS, Negrao-Correa D, de Figueiredo Antunes CM, Carneiro M (2009). Inaccuracy of enzyme-linked immunosorbent assay using soluble and recombinant antigens to detect asymptomatic infection by Leishmania infantum. PLoS Negl Trop Dis.

[45] Vaish M, Bhatia A, Reed SG, Chakravarty J, Sundar S (2012). Evaluation of rK28 antigen for serodiagnosis of visceral Leishmaniasis in India. Clin Microbiol Infect.

[46] Mukhtar M, Abdoun A, Ahmed AE, Ghalib H, Reed SG, Boelaert M, Menten J, Khair MM, Howard RF (2015). Diagnostic accuracy of rK28-based immunochromatographic rapid diagnostic tests for visceral leishmaniasis: a prospective clinical cohort study in Sudan. Trans R Soc Trop Med Hyg.

[47] da Silva MRB, Brandao NAA, Colovati M, de Sousa MMP, de Lima LC, Dorta ML, Ribeiro-Dias F, Costa DL, Costa CHN, de Oliveira MAP (2018). Performance of two immunochromatographic tests for diagnosis of visceral leishmaniasis in patients coinfected with HIV. Parasitol Res.

[48] Boelaert M, Rijal S, Regmi S, Singh R, Karki B, Jacquet D, Chappuis F, Campino L, Desjeux P, Le Ray D (2004). A comparative study of the effectiveness of diagnostic tests for visceral leishmaniasis. Am J Trop Med Hyg.

[49] Patil RR, Muliyil JP, Nandy A, Addy A, Maji AK, Chatterjee P (2012). Dynamics of the antibodies in cohorts of cured cases of visceral leishmaniasis: its implication on the validity of serological test, value in prognosis and in post therapeutic assessment. Hum Vaccin Immunotherapeutics.

[50] de Ruiter CM, van der Veer C, Leeflang MM, Deborggraeve S, Lucas C, Adams ER (2014). Molecular tools for diagnosis of visceral leishmaniasis: systematic review and meta-analysis of diagnostic test accuracy. J Clin Microbiol.

[51] Pizzuto M, Piazza M, Senese D, Scalamogna C, Calattini S, Corsico L, Persico T, Adriani B, Magni C, Guaraldi G (2001). Role of PCR in diagnosis and prognosis of visceral leishmaniasis in patients coinfected with human immunodeficiency virus type 1. J Clin Microbiol.

[52] Lachaud L, Dereure J, Chabbert E, Reynes J, Mauboussin JM, Oziol E, Dedet JP, Bastien P (2000). Optimized PCR using patient blood samples for diagnosis and follow-up of visceral Leishmaniasis, with special reference to AIDS patients. J Clin Microbiol.

[53] de Paiva-Cavalcanti M, de Morais RC, Pessoa ESR, Trajano-Silva LA, Goncalves-de-Albuquerque Sda C, Tavares Dde H, Brelaz-de-Castro MC, Silva Rde F, Pereira VR (2015). Leishmaniases diagnosis: an update on the use of immunological and molecular tools. Cell Biosci.

[54] Pourabbas B, Ghadimi Moghadam A, Pouladfar G, Rezaee Z, Alborzi A (2013). Quantification of leishmania infantum kinetoplast DNA for monitoring the response to meglumine antimoniate therapy in visceral leishmaniasis. Am J Trop Med Hyg.

[55] Albuquerque LC, Mendonca IR, Cardoso PN, Baldacara LR, Borges MR, Borges Jda C, Pranchevicius MC (2014). HIV/AIDS-related visceral leishmaniasis: a clinical and epidemiological description of visceral leishmaniasis in northern Brazil. Rev Soc Bras Med Trop.

[56] Nascimento ET, Moura ML, Queiroz JW, Barroso AW, Araujo AF, Rego EF, Wilson ME, Pearson RD, Jeronimo SM (2011). The emergence of concurrent HIV-1/AIDS and visceral leishmaniasis in Northeast Brazil. Trans R Soc Trop Med Hyg.

[57] RangelI O, Hiramoto RM, Henriques LF, Taniguchi HH, Ciaravolo RMC, Tolezano JE, França ACC, Yamashiro J, Oliveira SS (2013). Epidemiological classification of cities according to the Program of Surveillance and Control of American Visceral Leishmaniasis in the State of São Paulo, updated in 2013. BEPA Bol Epidemiol Paul.

[58] Brazil: Ministry of Health (2016). Department of Health Surveillance. Aids and STI. Brazil. Ministry of Health, editor. Epidemiological bulletin [in Portuguese].

